# Role of Heterogeneous Macromolecular Crowding and Geometrical Irregularity at Central Excitatory Synapses in Shaping Synaptic Transmission

**DOI:** 10.1371/journal.pone.0167505

**Published:** 2016-12-01

**Authors:** Rahul Gupta, Melissa Reneaux, Karmeshu

**Affiliations:** 1 School of Computational and Integrative Sciences, Jawaharlal Nehru University, New Delhi, India 110067; 2 School of Computer and Systems Sciences, Jawaharlal Nehru University, New Delhi, India 110067; Augusta University, UNITED STATES

## Abstract

Besides the geometrical tortousity due to the extrasynaptic structures, macromolecular crowding and geometrical irregularities constituting the cleft composition at central excitatory synapses has a major and direct role in retarding the glutamate diffusion within the cleft space. However, the cleft composition may not only coarsely reduce the overall diffusivity of the glutamate but may also lead to substantial spatial variation in the diffusivity across the cleft space. Decrease in the overall diffusivity of the glutamate may have straightforward consequences to the glutamate transients in the cleft. However, how spatial variation in the diffusivity may further affect glutamate transients is an intriguing aspect. Therefore, to understand the role of cleft heterogeneity, the present study adopts a novel approach of glutamate diffusion which considers a gamma statistical distribution of the diffusion coefficient of glutamate (*D*_*glut*_) across the cleft space, such that its moments discernibly capture the dual impacts of the cleft composition, and further applies the framework of superstatistics. The findings reveal a power law behavior in the glutamate transients, akin to the long-range anomalous subdiffusion, which leads to slower decay profile of cleft glutamate at higher intensity of cleft heterogeneity. Moreover, increase in the cleft heterogeneity is seen to eventually cause slower-rising excitatory postsynaptic currents with higher amplitudes, lesser noise, and prolonged duration of charge transfer across the postsynaptic membrane. Further, with regard to the conventional standard diffusion approach, the study suggests that the effective *D*_*glut*_ essentially derives from the median of the *D*_*glut*_ distribution and does not necessarily need to be the mean *D*_*glut*_. Together, the findings indicate a strong implication of cleft heterogeneity to the metabolically cost-effective tuning of synaptic response during the phenomenon of plasticity at individual synapses and also provide an additional factor of variability in transmission across identical synapses.

## Introduction

The phenomenon of synaptic transmission at central excitatory synapses plays an extremely crucial role in the functioning of the central nervous system. The various microscopic events that occur at the chemical synapses together facilitate the transmission of action potentials from one neuron to the other at the synaptic junctions [1]. Among these events, the diffusion of presynaptically-released glutamate in the cleft has a pivotal contribution to the synaptic activity as it crucially regulates the generation of excitatory postsynaptic currents (EPSCs) [2–4]. However, despite being a very prominent and biophysically fundamental process, much still remains unknown about the various factors which shape the glutamate transients in the synaptic cleft.

The highly retarded diffusion of glutamate in the cleft space is well known [5–7]. The factors involved in the retarded diffusion are strictly related with the structural features of the synaptic as well as extrasynaptic space. With regard to the features associated with synaptic space, there exists a large body of literature which strongly indicates that the synaptic clefts at central excitatory synapses are not simply free spaces but have a very complex macromolecular organization [8–10]. Moreover, experimental observations regarding the cleft morphology suggest that, rather than having a perfectly regular geometry, the cleft space possesses numerous sudden geometrical irregularities [11, 12]. These features directly and constantly hinder the entire passage of a diffusing glutamate molecule across the cleft space. On the other hand, the presence of extrasynaptic structures including partially-reflecting glial surfaces restricts the escape of glutamate from the cleft space at the cleft periphery. Accordingly, the resulting extrasynaptic geometrical tortousity slows down the glutamate diffusion within the cleft space in a feedback manner [4, 6, 13, 14].

Further, the macromolecular crowding and geometrical irregularity within the cleft space may mark a dual impact on the glutamate diffusion. First, they coarsely reduce the overall diffusivity of glutamate in the cleft space. Second, the spatial heterogeneity in crowding and geometrical irregularity may further cause substantial spatial variation in the glutamate diffusivity across the cleft space. A conventional way to physically realize the glutamate diffusion within the cleft space is implementation of the standard diffusion framework with an appropriately reduced effective diffusion coefficient of glutamate (*D*_*glut*_) [4, 15–18]. The reduced *D*_*glut*_ collectively acknowledges the various factors causing the obstructed diffusion of glutamate. However, it is important to note that this approach inherently ends up with the simplifying assumption that the resultant hindrance to glutamate diffusion is homogeneously prevailing in the cleft space. As a consequence, it is not able to precisely discern the dual ways through which the macromolecular crowding and geometrical irregularity may influence glutamate diffusion.

Therefore, under the naturally occurring situation, although the effect of extrasynaptically-originating geometrical tortousity on the glutamate diffusion would remain unaltered, the spatially-heterogeneous cleft composition may have additional significant effects on the glutamate transients and the resultant EPSC generation, which otherwise may not be captured through the standard diffusion framework with a reduced effective *D*_*glut*_. Motivated by these facts, the present study adopts an approach which may not only capture the reduction in overall diffusivity of the glutamate but may also respond to the spatial variation in glutamate diffusivity arising from the cleft heterogeneity. This approach involves a statistical distribution of *D*_*glut*_ across the cleft space capturing the above-mentioned dual impacts of heterogeneous cleft composition and employs the well-established framework of superstatistics [19, 20]. Glutamate diffusion within the cleft space is examined analytically as well as numerically through simulation of the Brownian dynamics of glutamate. Subsequently, the effect of resulting glutamate transients on the generation of EPSCs is examined. The results demonstrate that the consideration of cleft heterogeneity leads to power law behavior in the glutamate transients, akin to the sub-diffusive anomalous diffusion, which has further significant effects on the generated EPSCs.

## Materials and Methods

### CA1 synapse model

**Fig 1:**
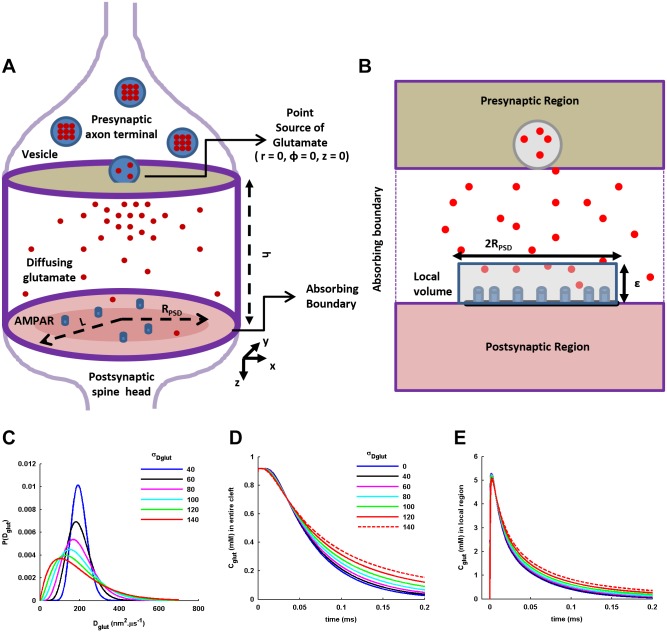
A schematic diagram of the simplified geometry of CA1 synapse, the statistical distribution of D_glut_ across cleft volume, and glutamate transients within the cleft. (A) A simplified cylindrical geometry of a CA1 synapse is shown. The perfectly absorbing cylindrical wall at a radial distance of *L* imitates the glutamate uptake by the transporters located on the glial membrane surrounding the cleft space. The upper and lower reflecting circular faces represent the presynaptic and postsynaptic membranes of the synapse, respectively, separated by a distance *h* which depicts the height of the cleft space. A concentric circular area on the postsynaptic membrane of radius *R*_*PSD*_ represents the PSD where AMPARs are located at high density. (B) A small concentric cylindrical volume of radius *R*_*PSD*_ and height *ε* above the PSD refers to the local region in which the glutamate transient causes activation of AMPARs located within the PSD. (B) The gamma statistical distributions of *D*_*glut*_ across the cleft space with fixed mean *D*_*glut*_, <*D*_*glut*_ > = 200*nm*^2^.*μs*^−1^, and varying standard deviations, *σ*_*Dglut*_, in *nm*^2^.*μs*^−1^. (C) and (D) show the spatially-averaged temporal profiles of glutamate concentration, *C*_*glut*_(*t*), in the entire cleft space and the local region above the PSD, respectively, for different values of *σ*_*Dglut*_.

The cleft at a CA1 synapse located on the average-sized mushroom or stubby spine on the dendrite of CA1 pyramidal neuron [21] is modeled as a cylindrical space (Fig 1A) [4, 16]. Its absorbing cylindrical wall imitates the glutamate uptake by the transporters present on the glial membrane surrounding the cleft. Partial reflection of glutamate from the glial membrane is neglected as it helps in filtering out its potential effect on the glutamate decay profile [6] and helps in examining only the role of diffusion within the cleft in shaping the decay profile under the spatially-distributed cleft *D*_*glut*_. On the other hand, the upper and lower reflecting circular faces represent the presynaptic and postsynaptic membranes of the synapse, respectively. The radial size of the cleft is kept 240*nm*, which equals to an area of 0.1810*μm*^2^ close to the synapse patch-area on an average-sized mushroom spine [11, 12]. The cleft height is kept 20*nm* [4, 6]. Any glutamate uptake inside the cleft space is not considered [18].

A synaptic vesicle on the presynaptic side contains 2000 glutamate molecules. For an average diameter of 35*nm* of the vesicle [22], the glutamate concentration in the vesicle equals 197.22*mM*, in the range 60-210*mM* observed experimentally [23]. In the present study, only uni-quantal synaptic events are considered. Majority of average-sized CA1 synapses possess single active zone and most release events at the synapse involve single vesicle release [12, 24]. For simplicity, the release of synaptic vesicle is performed here only at the center of the presynaptic face, which is also the center of the active zone right above the PSD (Fig 1A). The release of total glutamate content from the vesicle is assumed to occur instantaneously (a point source release) [17]. This setup involving an instantaneous release of 2000 glutamate molecules only at the center of the presynaptic face constitutes the main framework of the present study. Later on, the transmitter content and radial position of the release site would also be varied to examine the impact of cleft heterogeneity on EPSC generation.

The radius of PSD (*R*_*PSD*_) is taken 100*nm*, which is the typical size of the hot-spot of AMPARs activation right below the point of vesicle release (Fig 1A) [18, 25]. Also, the corresponding area of PSD equals to 0.0314*μm*^2^ which is the average area of PSD at non-perforated CA1 synapses measured experimentally [11, 12]. 30 AMPARs are uniformly located in the PSD [26]. Generation of EPSC only from the AMPARs located within the PSD is considered as it constitutes the major fraction (almost 80%) of EPSC generated at the synapse [18]. For the activation of AMPARs, glutamate transient in a local cylindrical volume right above the PSD (Fig 1B) is considered which is concentric with the cleft cylindrical volume but has a radius equals to *R*_*PSD*_ and height 5*nm* [4, 16].

### Statistical Distribution of *D*_*glut*_ across cleft

Instead of considering an exact spatial profile of *D*_*glut*_ variation (*D*_*glut*_ as a function of location) in the cleft, we consider the statistical distribution of spatially-varying magnitudes of cleft *D*_*glut*_. This statistical distribution portrays the abundance of slow- and fast-diffusion microdomains present in the cleft space. Each microdomain has a certain spatial spread within which the *D*_*glut*_ does not vary over a certain timescale which is much larger than that of the glutamate diffusion [19]. The consideration of the statistical distribution of *D*_*glut*_ allows the framework of superstatistics [19, 20] to work in the context of glutamate diffusion. In this regard, any sufficient experimental estimate is not presently available. However, studies performed on other physical systems involving diffusion in crowded and disordered medium [27–30] may prove helpful in deciding the appropriate form of the statistical distribution of *D*_*glut*_ possibly present in the cleft. Accordingly, a gamma-distribution of *D*_*glut*_ is considered across the cleft space (Fig 1C). The probability distribution function *P*(*D*_*glut*_) is defined as

$$\begin{array}{c} P\left(D_{glut}\right)=\frac{1}{\Gamma \left(k\right)\theta^{k}}{D_{glut}}^{k-1}\exp\left(-\frac{D_{glut}}{\theta }\right) \end{array}$$ (1)

where, *k* and *θ* are the shape and scale parameters, respectively, of the gamma distribution. The mean and standard deviation of the distribution are defined as

$$\begin{array}{c} <D_{glut}>=k\theta \end{array}$$ (1a)

$$\begin{array}{c} \sigma_{Dglut}=\sqrt{k\theta^{2}} \end{array}$$ (1b)

Here, the <*D*_*glut*_> signifies the retarded diffusion of glutamate assuming a perfectly homogenously crowded cleft medium and also includes the effect of extrasynaptically-originating geometrical tortousity. Therefore, <*D*_*glut*_> is kept fixed at 200*nm*^2^.*μs*^−1^, in the range 100-400*nm*^2^.*μs*^−1^ of *D*_*glut*_ observed in the synaptic cleft [5, 7]. Only the *σ*_*Dglut*_ is varied to examine the effect of cleft heterogeneity on the synaptic response. This is done by arbitrarily increasing the *σ*_*Dglut*_, where six sample points of *σ*_*Dglut*_ (40, 60, 80, 100, 120 and 140 *nm*^2^.*μs*^−1^) with a regular interval of 20*nm*^2^.*μs*^−1^ are taken (Fig 1C). As evident, a broad range of the coefficient of variation, CV, (0.2-0.7) for the *D*_*glut*_ distribution is tested. *σ*_*Dglut*_ = 0*nm*^2^.*μs*^−1^ corresponds to the conventional diffusion with the fixed <*D*_*glut*_>, neglecting the spatial fluctuations in <*D*_*glut*_>. On the other hand, *σ*_*Dglut*_ = 20*nm*^2^.*μs*^−1^ is not examined as the corresponding shape parameter k grows to a very large value of 100 where the gamma-distribution approaches the Gaussian-distribution and the *P*(*D*_*glut*_) is not able to be suitably defined by the given gamma distribution. In fact, such a small *σ*_*Dglut*_ (CV = 0.1) may eventually account to the situation of conventional diffusion only.

### Spatiotemporal profile of glutamate concentration in cleft

According to the analytical approach, the point release of vesicle transmitters in the cylindrical cleft space is considered to occur at the time point *t* = 0 and the spatial location *r* = 0 and *z* = 0, where *r* denotes the radial distance and *z* denotes the height in the positive direction. The temporal evolution of glutamate concentration at any given spatial location (*r*, *z*) in the cleft, under the simplifying assumption of radial symmetry, is given by

$$\begin{array}{c} \frac{\partial C(r,z,t)}{\partial t}=D_{glut}\left\{\frac{\partial^{2}}{\partial r^{2}}+\frac{1}{r}\frac{\partial }{\partial r}+\frac{\partial^{2}}{\partial z^{2}}\right\}C\left(r,z,t\right) \end{array}$$ (2)

with the initial condition as well as absorbing and reflecting boundary conditions defined as

$$\begin{array}{c} C\left(r,z,t=0\right)=\frac{N_{T}}{4\pi r}\delta \left(r\right)\delta \left(z\right) \end{array}$$ (2a)

$$\begin{array}{c} C(r=L,z,t)=0 \end{array}$$ (2b)

$$\begin{array}{c} \frac{\partial C(r,z=0,t)}{\partial z}=\frac{\partial C(r,z=h,t)}{\partial z}=0 \end{array}$$ (2c)

Here, *L* and *h* denote the radius and height of the cleft space, respectively. *N*_*T*_ denotes the total number of transmitter molecules released from the vesicle fusion. Consequently, the solution of the above diffusion equation under the prescribed initial and boundary conditions provides us the spatiotemporal profile of glutamate concentration, which is given by

$$\begin{array}{c} C\left(r,z,t\right)=\frac{2N_{T}}{\pi L^{2}h}\sum_{n=1}^{+\infty }\sum_{m=-\infty }^{+\infty }\frac{J_{0}\left(k_{n}r\right)}{J_{1}^{2}\left(\lambda_{0n}\right)}\cos\left(\eta_{m}z\right)e^{-\left(k_{n}^{2}+\eta_{m}^{2}\right)D_{glut}t} \end{array}$$ (3)

$$\begin{array}{c} k_{n}=\frac{\lambda_{0n}}{L},\;n=1,2,3... \end{array}$$ (3a)

$$\begin{array}{c} \eta_{m}=\frac{m\pi }{h},\;m=-\infty ,...,-1,0,1,...,+\infty \end{array}$$ (3b)

*J*_0_ and *J*_1_ are the Bessel functions of 0^*th*^ and 1^*st*^ orders, respectively. *λ*_0*n*_ denotes the *n*^*th*^ root of the *J*_0_. The expression *C*(*r*, *z*, *t*) describes the situation of conventional diffusion of glutamate with a fixed reduced *D*_*glut*_ in a perfectly homogenously-crowded cleft medium.

However, when we are dealing with diffusion in the presence of distributed *D*_*glut*_, *C*(*r*, *z*, *t*) becomes a conditional expression given a random value of *D*_*glut*_ i.e. *C*(*r*, *z*, *t*|*D*_*glut*_). Accordingly, following the principles of superstatistics [19, 20], the spatiotemporal profile of glutamate under the heterogeneously-crowded cleft condition, *C*^*rnd*^(*r*, *z*, *t*), can be effectively approximated by integrating *C*(*r*, *z*, *t*) obtained with a fixed *D*_*glut*_ over the probability distribution of the *D*_*glut*_.

$$\begin{array}{c} C^{rnd}\left(r,z,t\right)=\int_{0}^{+\infty }C\left(r,z,t|D_{glut}\right)P\left(D_{glut}\right)dD_{glut} \end{array}$$ (4)

$$\begin{array}{c} \Rightarrow C^{rnd}\left(r,z,t\right)=\frac{1}{\theta^{k}}\frac{2N_{T}}{\pi L^{2}h}\sum_{n=1}^{+\infty }{\sum_{m=-\infty }^{+\infty }\frac{J_{0}\left(k_{n}r\right)}{J_{1}^{2}\left(\lambda_{0n}\right)}\cos\left(\eta_{m}z\right)\left(\left(k_{n}^{2}+\eta_{m}^{2}\right)t+\frac{1}{\theta }\right)}^{-k} \end{array}$$ (5)

The power law behavior *C*^*rnd*^(*r*, *z*, *t*)∼*t*^−*k*^ indicates a long-range anomalous sub-diffusion of glutamate in the cleft. In fact, the expression is a first-order approximation to the results of anomalous diffusion typically obtained from the continuous time random walk model and the fractional diffusion framework. Next, we compute a general expression for the temporal profile of glutamate in a coaxial cylindrical volume within the cleft with a radius *ρ* (such that *ρ* ≤ *L*) and height *ε* (i.e. [*h* − *ε*, *h*]). On spatially averaging the expressions *C*(*r*, *z*, *t*) and *C*^*rnd*^(*r*, *z*, *t*) over the desired volume, we obtain the temporal profiles of glutamate under the two conditions of diffusion as

$$\begin{array}{ccc} C(t) & = & \frac{2N_{T}}{\pi L\rho \varepsilon }\sum_{n=1}^{+\infty }\sum_{m=-\infty .m\neq 0}^{+\infty }\frac{J_{1}\left(k_{n}\rho \right)}{\lambda_{0n}J_{1}^{2}\left(\lambda_{0n}\right)}\frac{{\left(-1\right)}^{m}}{m\pi }\sin\left(\eta_{m}\varepsilon \right)e^{-(k_{n}^{2}+\eta_{m}^{2})Dt}\\ & & +\frac{2N_{T}}{\pi L\rho h}\sum_{n=1}^{+\infty }\frac{J_{1}\left(k_{n}\rho \right)}{\lambda_{0n}J_{1}^{2}\left(\lambda_{0n}\right)}e^{-(k_{n}^{2}Dt)} \end{array}$$ (6)

$$\begin{array}{ccc} C^{rnd}\left(t\right) & = & \frac{2N_{T}}{\pi L\rho \varepsilon \theta^{k}}\sum_{n=1}^{+\infty }\sum_{m=-\infty ,m\neq 0}^{+\infty }\frac{J_{1}\left(k_{n}\rho \right)}{\lambda_{0n}J_{1}^{2}\left(\lambda_{0n}\right)}\frac{{\left(-1\right)}^{m}}{m\pi }\sin\left(\eta_{m}\varepsilon \right){\left(\left(k_{n}^{2}+\eta_{m}^{2}\right)t+\frac{1}{\theta }\right)}^{-k}\\ & & +\frac{2N_{T}}{\pi L\rho h\theta^{k}}\sum_{n=1}^{+\infty }\frac{J_{1}\left(k_{n}\rho \right)}{\lambda_{0n}J_{1}^{2}\left(\lambda_{0n}\right)}{\left(k_{n}^{2}t+\frac{1}{\theta }\right)}^{-k} \end{array}$$ (7)

If we keep *ρ* = *L* and *ε* = *h* in the above expressions, *C*(*t*) and *C*^*rnd*^(*t*) provide the temporal profiles of glutamate in the entire cleft (Fig 1A). On the other hand, if we keep *ρ* = *R*_*PSD*_ and *ε* = 5*nm*, *C*(*t*) and *C*^*rnd*^(*t*) provide the temporal profiles of glutamate concentration in the thin local volume right above the PSD which causes the activation of AMPARs (Fig 1B). For convenience, the *C*(*t*) and *C*^*rnd*^(*t*) are together referred to as *C*_*glut*_(*t*) in the following content.

According to the numerical approach, at the time of point-release of transmitters from the vesicle, each transmitter molecule is randomly assigned a diffusion coefficient from the gamma-distribution of *D*_*glut*_ with the fixed <*D*_*glut*_> and a chosen *σ*_*Dglut*_ of interest. Subsequently, a Brownian dynamics of the transmitters is simulated [4, 16, 17] with the absorbing and reflecting boundary conditions mentioned under the analytical approach. The time-step of Brownian simulation is kept 0.1*μs*. Following an identical procedure, 600 such events of transmitter diffusion are independently performed and the sample temporal profiles of the glutamate concentration in the local volume above the PSD are recorded for a time span of 4*ms*.

### Activation of AMPARs and generation of EPSCs

The activation of AMPARs located in the PSD is performed by the temporal profile of glutamate concentration in the thin local volume above the PSD. Here, it is assumed that all the AMPARs sense almost the same glutamate concentration in the local volume [31]. The Milstein-Nicoll scheme of AMPA receptor activation is used [32]. In the analytical treatment, the dynamical equation of receptor activation is solved using forward Euler-integration with a time-step of 0.1*μs* and the glutamate-dependent transition-rate is governed by the analytically-obtained *C*_*glut*_(*t*). The total open-state population of the AMPARs is considered which comprises both the two and three glutamate-bound open-state populations. Opening of each receptor contributes, on average, 10.1*pS* conductance [18] to the post-synaptic membrane for the generation of EPSC.

For the stochastic simulations of AMPARs activation in response to the 600 individual events of transmitter release (as mentioned above), the modified Gillespie algorithm [33] with minimum time-step correction of 0.1*μs* is used. In each release event, the transition-rate of AMPAR activation is dependent on the temporal profile of glutamate obtained from the Brownian dynamics simulation. Subsequently, the sample temporal profiles of total-open state population of AMPARs are used to generate the temporal profiles of EPSCs.

All the numerical simulations have been performed using the software MATLAB (The MathWorks, Natick) and the codes are posted on ModelDB (http://senselab.med.yale.edu/ModelDB/showModel.cshtml?model=206328).

## Results

### Glutamate transients within synaptic cleft

To investigate the impact of cleft heterogeneity mediated through the setup of a distributed *D*_*glut*_ on the glutamate transient in the cleft, we observe the spatially-averaged temporal profile of glutamate concentration, *C*_*glut*_(*t*), in the entire cleft space (Fig 1D) as well as in the local volume (Fig 1E) right above the PSD (Fig 1B), using Eqs (6) and (7). The reason behind additionally observing *C*_*glut*_(*t*) in the local volume is that, instead of the *C*_*glut*_(*t*) in entire cleft space, it is the local *C*_*glut*_(*t*) which essentially causes the glutamate-dependent activation of AMPARs [4, 16]. It is seen that, under all conditions of *σ*_*Dglut*_, the peak *C*_*glut*_(*t*) in the entire cleft space remains ∼ 0.9-1.0*mM*, which is consistent with the peak glutamate concentration in the cleft observed in the earlier studies [2, 5, 18, 34]. However, the local concentration near the PSD is higher, ∼ 5-6*mM*, due to the substantially small volume of the local region above AMPARs.

**Fig 2:**
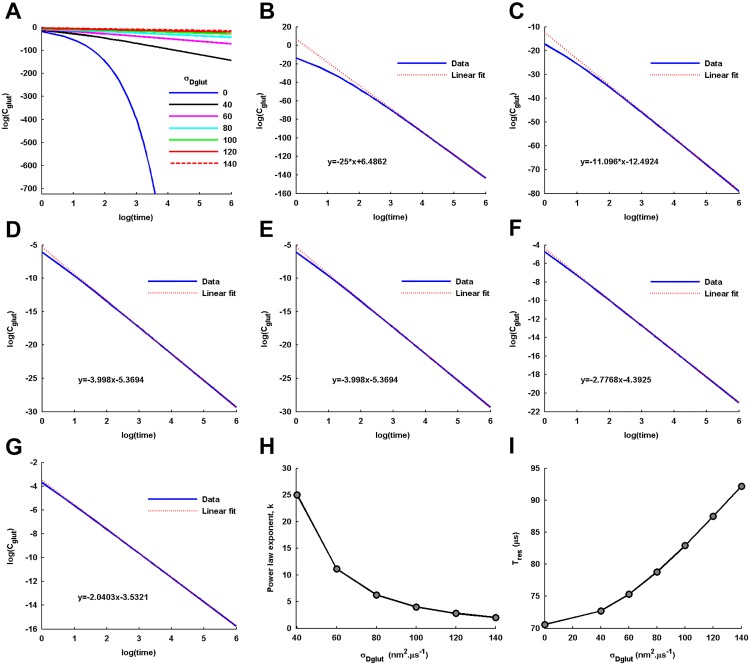
Power law behavior in glutamate transients. (A) A relative comparison of log-log plots of *C*_*glut*_(*t*) (in M units) over long time intervals of 4*ms* for glutamate diffusion with the different values of *σ*_*Dglut*_. The diffusion with a non-zero *σ*_*Dglut*_ depicts a linear log-log tail of decay over long time, a prominent feature of power law phenomenon, whereas that under the standard diffusion with zero *σ*_*Dglut*_ rapidly decreases in a nonlinear manner. (B-G) Linear fittings to the log-log plots for the non-zero values of *σ*_*Dglut*_, along with their fitting constants. The slope of the linear fitting provides the power law exponent, *k*. (H) The profile of variation in *k* with change in *σ*_*Dglut*_ demonstrates the rapid slowing down of the *C*_*glut*_(*t*) decay profile with the increase in *σ*_*Dglut*_. (I) The associated increase in the mean residence time, *T*_*res*_, of a glutamate molecule in the cleft with the increase in *σ*_*Dglut*_.

In the presence of non-zero *σ*_*Dglut*_, the log-log plot of *C*_*glut*_(*t*) over long time depicts a power law behavior in glutamate transient, *C*_*glut*_(*t*)∼*t*^−*k*^, which is strikingly different from that of the *C*_*glut*_(*t*) under standard diffusion performed with only <*D*_*glut*_> (Fig 2A). The linear fittings to the log-log plots for the different non-zero values of *σ*_*Dglut*_ (Fig 2B–2G) demonstrate that the power law exponent *k* of the glutamate transient is inversely proportional to the *σ*_*Dglut*_ (Fig 2H). Accordingly, increase in *σ*_*Dglut*_ leads to slower decay profile of *C*_*glut*_(*t*) and consequently increases the mean residence time (*T*_*res*_) of glutamate in the cleft (Fig 2I).

**Fig 3:**
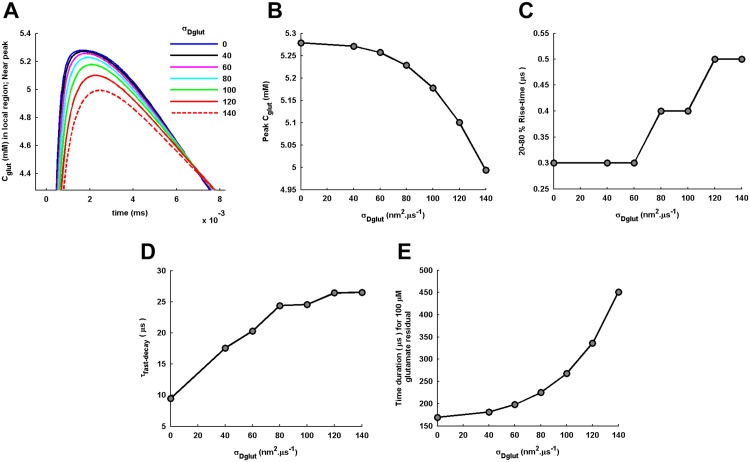
Amplitude and temporal characteristics of local glutamate transients. (A) A zoomed-in view of the local *C*_*glut*_(*t*) around its peak level portrays the effect of variation in *σ*_*Dglut*_ on the amplitude and rise-time of the local *C*_*glut*_(*t*). (B) Increase in *σ*_*Dglut*_ causes reduction in the peak level of local *C*_*glut*_(*t*). (C) The concomitant increase in 20-80% rise-time of local *C*_*glut*_(*t*) demonstrates slowing down of the accumulation of glutamate concentration in the local region above PSD. (D) The increase in *τ*_*fast*–*decay*_ with increase in *σ*_*Dglut*_ shows the slowing down of the early decay profile of the local *C*_*glut*_(*t*). (E) Increase in the time duration, since the point of vesicle fusion, by which the local *C*_*glut*_(*t*) decays to less than 100*μM* concentration effectively demonstrates the prolongation of the glutamate residual of low concentration in the cleft.

A closer look into the local *C*_*glut*_(*t*) around the peak concentration (Fig 3A) shows that the peak concentration as well as the rise-time are also affected by the variation in *σ*_*Dglut*_. We observe a gradual decrease in the peak level of local *C*_*glut*_(*t*) (Fig 3B) and an almost sigmoidal increase in its 20-80% rise-time with increase in the *σ*_*Dglut*_ (Fig 3C). The sigmoidal increase in rise time depicts that, for the given <*D*_*glut*_>, there exists a critical *σ*_*Dglut*_ around which a sudden inflection in the rise-time occurs.

The biphasic decay profile of *C*_*glut*_(*t*) in the entire cleft space as well as in the local region is clearly evident under all conditions of diffusion (Fig 1D and 1E). An important point to note here is that the presence of *σ*_*Dglut*_ affects the decay profile of *C*_*glut*_(*t*) through the power law behavior not only over long time-trail but also through slowing down of the early decay profile, which is extremely relevant to the AMPAR activation. The time-constant of the early decay profile computed from the local *C*_*glut*_(*t*) is seen to significantly increase with the increase in *σ*_*Dglut*_ (Fig 3D). The observation regarding the early decay profile is consistent with the previous studies suggesting that the initial rapid decay in glutamate transients in the cleft is primarily governed by the conditions of glutamate diffusion within the cleft [2, 34].

At the same time, the earlier studies have also shown that the later long-trails of low concentrations (in 100*μM* range) of the glutamate transients are mainly regulated by presence of the extrasynaptic structures including partially-reflecting glial membrane and glial transporter density [6, 34–38]. However, the present study suggests that the long-time trail of cleft glutamate may also involve significant contribution from the power law behavior of glutamate diffusion arising from the diffusion conditions within the cleft. To demonstrate this, we record the time point by which the local *C*_*glut*_(*t*) in the cleft is reduced to almost 100*μM* concentration (Fig 3E). The result clearly shows that the power law behavior significantly extends the time interval of the glutamate residual in *μM* range.

**Fig 4:**
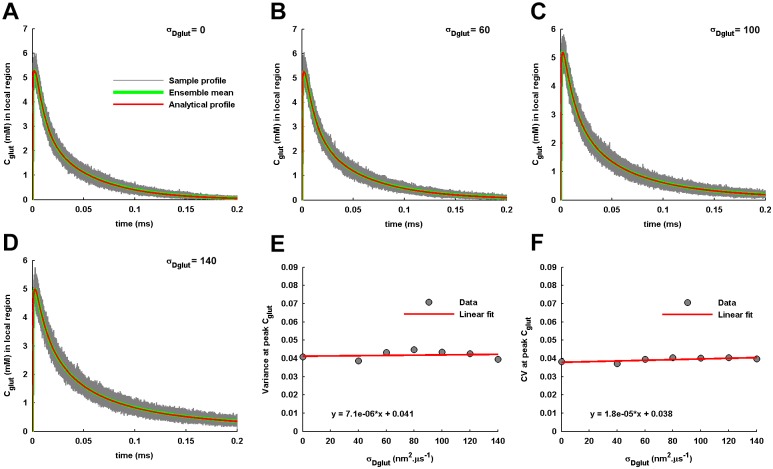
Variability in local glutamate transients across independent events of single vesicle-fusion. (A) 150 sample temporal profiles of local *C*_*glut*_(*t*) under the independent events of vesicle-fusion at the center of the presynaptic face and with the identical transmitter content of 2000 glutamate molecules are shown for the standard diffusion condition with zero *σ*_*Dglut*_. The sample profiles are obtained by the numerical simulation of the Brownian dynamics of glutamate molecules in the cleft. The ensemble mean across 600 such sample temporal profiles identically matches the analytically-obtained local *C*_*glut*_(*t*). (B-D) provide a similar observation for the diffusion performed with the non-zero values of *σ*_*Dglut*_. (E) The linear fitting to the variances of local *C*_*glut*_(*t*) at its peak level under different conditions of *σ*_*Dglut*_ demonstrate a very slow (see the slope of the fitting provided) increase in the amount of fluctuation around the peak local *C*_*glut*_(*t*) with increase in *σ*_*Dglut*_. (F) Similar to the variance, the CV of *C*_*glut*_(*t*) at its peak level also shows a very gradual increase with the increase in *σ*_*Dglut*_.

Further, the numerical simulation of the Brownian dynamics of glutamate in cleft for an individual *σ*_*Dglut*_ provides an ensemble of sample temporal profiles of glutamate concentration in the local volume recorded for 600 independent release events (Fig 4A-4D). The mean temporal profile of concentration is obtained by averaging over the ensemble of sample profiles. It is observed that the mean temporal profile always matches with the analytically obtained *C*_*glut*_(*t*) for individual values of *σ*_*Dglut*_. Since glutamate transient in the local volume acts like an activation signal for AMPARs located in the PSD, the effect of distributed *D*_*glut*_ on the noise in local glutamate signal is examined through computation of the variance (Fig 4E) and the CV (Fig 4F) at the peak *C*_*glut*_(*t*). Interestingly, both the variance and the CV are almost unaffected with the increase in *σ*_*Dglut*_.

As a consequence, it is revealed that the cleft heterogeneity affects the glutamate transients in the cleft by predominantly affecting their amplitudes and temporal characteristics. Increase in *σ*_*Dglut*_ of the gamma-distributed *D*_*glut*_ across the cleft space reduces the amplitude of glutamate transients and delays their rising as well as decaying profiles. These effects of cleft heterogeneity on the glutamate transients can further lead to substantial alterations in the resulting generation of postsynaptic EPSCs through AMPARs activation.

### Effect of glutamate transients on the temporal profiles of EPSCs

**Fig 5:**
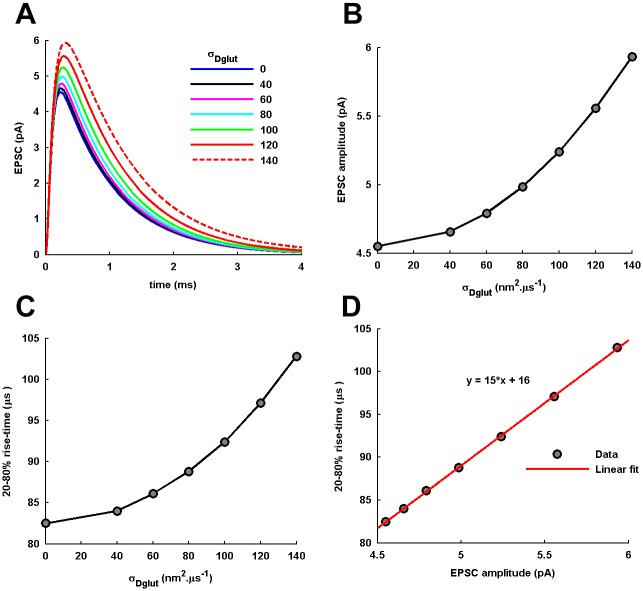
Temporal profiles of EPSC generation. (A) The analytically-obtained temporal profiles of postsynaptic EPSC generation (the *negative* sign of inward current has been inverted) by the activation of AMPARs located within the PSD in response to the analytically-obtained local *C*_*glut*_(*t*) under the different conditions of *σ*_*Dglut*_. (B) The EPSC amplitude and (C) the 20-80% rise-time of the EPSC profile are seen to increase with the increase in *σ*_*Dglut*_. (D) The variations in the EPSC amplitude and 20-80% rise time demonstrate a strong positive correlation as observed from the linear fitting.

Next, we examine the consequences of the power law behavior in the local *C*_*glut*_(*t*) arising from cleft heterogeneity to the generation of postsynaptic EPSCs. A very prominent effect of increasing *σ*_*Dglut*_ can be directly seen in the prolongation of the AMPARs activation (Fig 5A). Another effect is observed through the gradual rise in the EPSC amplitudes with the increase in *σ*_*Dglut*_ (Fig 5B). Both these effects are the consequences of the concomitant slowing down of the early decay profile of local *C*_*glut*_(*t*) (Fig 3D) as well as increase in the overall *T*_*res*_ of glutamate (Fig 2I), due to the more pronounced power law behavior in *C*_*glut*_(*t*) (Fig 2A). Further, the 20-80% rise-time of EPSC profiles also considerably increases (Fig 4C), owing to the slower rising profile of local *C*_*glut*_(*t*) (Fig 3C) and decrease in the peak *C*_*glut*_(*t*) (Fig 3B).

Here, a remarkable feature is the strong positive correlation between the variations in the EPSC amplitude and rise-time under varying *σ*_*Dglut*_ (Fig 5D). In the literature, slower-rising EPSC profiles with higher amplitudes are known to occur predominantly through two kinds of phenomena at a synapse viz. multi-vesicular release with small temporal jittering [18] and glutamate spill-out from neighboring synapses [5, 39]. Other ways to enhance the EPSC amplitude such as, through increase in glutamate content of the presynaptic vesicles [18, 40] or through prolonged glutamate release [5], have been shown to cause a simultaneous decrease in the 20-80% rise-time of EPSCs. Therefore, the manner in which the power law behavior in local *C*_*glut*_(*t*) affects the EPSC profile under varying conditions of *σ*_*Dglut*_ but fixed <*D*_*glut*_> is very eccentric in nature and deserves a special note.

A recent detailed simulation study performed by Ventriglia [41] which involved diffusion of glutamate in the presence of trans-synaptic fibrils provides a supportive evidence for the role of cleft macromolecular crowding in shaping the EPSC amplitude, as illustrated here. Increasing the density of filaments was shown to enhance the EPSC amplitude due to the increase in the probability of AMPAR-glutamate interaction mediated through the underlying slowing of glutamate diffusion. Moreover, a small increase in the EPSC amplitude (5-20%) due to increase in the density of trans-synaptic filaments is similar to the small increment (almost 31%) in the EPSC amplitude demonstrated here (Fig 5B). The previous study mainly focused on the ultimate consequence of the cleft crowding i.e. on the EPSC generation and could not show in detail how the glutamate diffusion is affected in the presence of trans-synaptic fibrils acting as steric obstructions in the cleft. However, it has been suggested there that the reduction in free-diffusing space in cleft due to the presence of trans-synaptic fibrils, which is different from the tortuosity factor, is a potential reason behind slowing of the glutamate diffusion and enhancement of EPSC generation.

**Fig 6:**
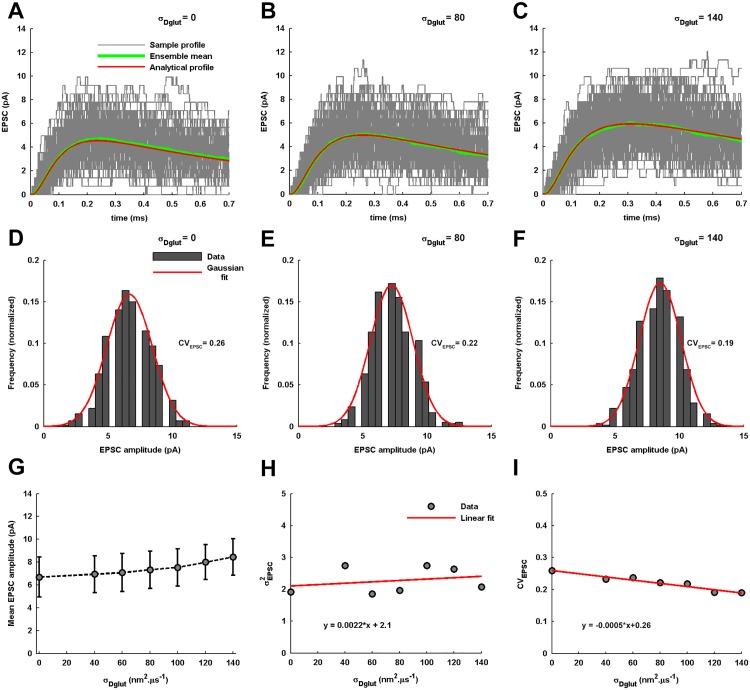
Variability in EPSC profile across independent events of single vesicle-release. (A) 150 sample temporal profiles of EPSC generation due to the stochastic activation of AMPARs located within the PSD in response to the numerically-obtained sample temporal profiles of local *C*_*glut*_(*t*) under the independent events of vesicle-fusion are shown for the standard diffusion condition with zero *σ*_*Dglut*_. The ensemble mean across 600 such sample temporal profiles closely matches the analytically-obtained temporal profile of EPSC. (B-C) provide a similar observation for the cases with the non-zero values of *σ*_*Dglut*_. (D-F) shows the distribution of EPSC amplitudes across the ensemble for the individual values of *σ*_*Dglut*_. The EPSC amplitudes are found to always exhibit a Gaussian distribution (fitting correlations; *r*^2^ = 0.9797 for *σ*_*Dglut*_ = 0, *r*^2^ = 0.9585 for *σ*_*Dglut*_ = 80, and *r*^2^ = 0.9663 for *σ*_*Dglut*_ = 140). The corresponding values of *CV*_*EPSC*_ are also mentioned. (G) The error-bar plot of variation in the mean EPSC amplitude with change in *σ*_*Dglut*_. The solid circles represent the mean EPSC amplitude whereas the vertical lines represent one standard deviation of the amplitude distribution around its mean. It is seen that the mean EPSC amplitude increases with the increase in *σ*_*Dglut*_. (H) and (I) show the linear fittings to the change in the variance, $\sigma_{EPSC}^{2}$, and *CV*_*EPSC*_ of the EPSC amplitude distribution with change in *σ*_*Dglut*_, respectively. $\sigma_{EPSC}^{2}$ is seen to very slowly increase with the increase in the *σ*_*Dglut*_ whereas the *CV*_*EPSC*_ gradually decreases (almost 27%) depicting an increase in the reliability of synaptic transmission.

Further, the stochastic simulations of AMPAR activation under independent trial-to-trial vesicle release events for an individual *σ*_*Dglut*_ provide an ensemble of 600 sample temporal profiles of EPSC generation (Fig 6A-6C). It is evident that the mean EPSC profile obtained by averaging over the ensemble closely resembles the analytically obtained EPSC profile for all values of *σ*_*Dglut*_. The EPSC amplitudes across the ensemble demonstrate a Gaussian distribution (Fig 6D-6F) and the mean EPSC amplitude is found to significantly increase with increase in *σ*_*Dglut*_ (Fig 6G). It is important to note here that the magnitudes of mean EPSC amplitudes are close to the mean amplitude (4.7-5.8*pA*) experimentally recorded for the smallest dendritic events at CA1 synapses [42].

On the other hand, the variance ($\sigma_{EPSC}^{2}$) of EPSC amplitude-distribution increases slowly with the increase in *σ*_*Dglut*_ (Fig 6H). Since *CV*_*EPSC*_ is the ratio of $\sqrt{\sigma_{EPSC}^{2}}$ to the mean EPSC amplitude, the sharp increase in the latter relative to the former leads to a gradual decrease in *CV*_*EPSC*_ under increasing *σ*_*Dglut*_ (Fig 6I). A decrease in *CV*_*EPSC*_ along with a substantial increase in EPSC amplitude eventually suggests that increase in the statistical fluctuation in *σ*_*Dglut*_ around its fixed <*D*_*glut*_> causes slower-rising EPSCs with higher amplitude, better reliability, and a prolonged duration.

### Dependency of cleft heterogeneity-mediated tuning of EPSC on the critical parameters of instantaneous transmitter release

As stated previously, a quantal release of glutamate into the synaptic cleft is the source of chemical signal for the activation of AMPARs. Therefore, an obvious question arises that how the two critical parameters of the instantaneous transmitter release viz. the quantity of glutamate released from a single vesicle-fusion (*N*_*T*_) and the radial location of the presynaptic site of vesicle-fusion (*r*_0_) may govern the impact of cleft heterogeneity on the various features of the EPSC generation. The set of synaptic parameters which has been employed for our previous opening investigation would then be called as the standard setup, for the convenience of repeatedly referring to it in the text. Therefore, with regard to the two specific parameters varied here, the standard setup involves *N*_*T*_ = 2000 and *r*_0_ = 0*nm*. While varying one of these parameters of interest, the rest of the parameters are kept locked to the standard setup. In this way, it enables us to untangle the independent roles of these specific critical parameters in regulating the action of cleft heterogeneity. Similar to the investigation involving standard setup, a two-fold approach is adopted here using analytical as well as numerical treatment. Throughout these investigations, the AMPAR density in the PSD is unaltered and is identical to that mentioned under the standard setup.

**Fig 7:**
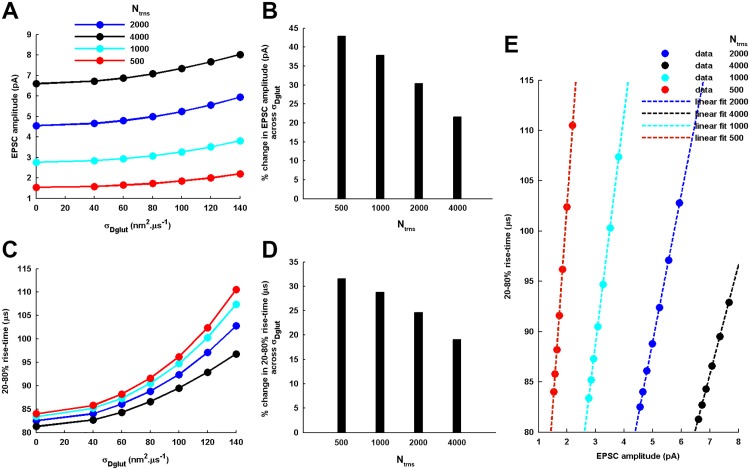
Dependency of the cleft heterogeneity-mediated tuning of EPSC amplitude and 20-80% rise-time on the presynaptically-released transmitter content, N_T_. Accordingly, the other parameters of the synapse model are kept identical to that considered under the previously-investigated standard framework. (A) and (C) demonstrate the effect of considering different *N*_*T*_ on the profile of increase in EPSC amplitude and 20-80% rise-time, respectively, across rising *σ*_*Dglut*_. Notably, the amplitude and rise-time profiles shift to higher and lower values, respectively, as the *N*_*T*_ is increased. (B) and (D) provide the percentage change in the EPSC amplitude and 20-80% rise-time, respectively, across the entire range of increasing *σ*_*Dglut*_ for the different values of *N*_*T*_. (E) demonstrates that the variations in EPSC amplitude and rise-time always follow a strict linear relationship for all values of *N*_*T*_ (linear fittings: *y* = 10.862 ∗ *x* + 9.6601 for *N*_*T*_ = 4000, *y* = 14.624 ∗ *x* + 15.918 for *N*_*T*_ = 2000, 22.849 ∗ *x* + 20.272 for *N*_*T*_ = 1000, and *y* = 40.11 ∗ *x* + 22.322 for *N*_*T*_ = 500) and, thus, depicts a characteristic property of the cleft heterogeneity-mediated tuning of the two EPSC features.

**Variation in N_T_.** The number of glutamate molecules instantaneously released from a single event of vesicle fusion is varied in multiples of the average count *N*_*T*_ = 2000 released at a CA1 synapse. *N*_*T*_ = 1000 and *N*_*T*_ = 4000 fall well within the typically observed range of glutamate concentration (60-210*mM*) in a vesicle [23]. However, release of an extremely low glutamate quantity, such as *N*_*T*_ = 500 examined here, may occur most probably through kiss-and-run partial vesicle fusion events [1]. The results obtained from the analytical treatment (Fig 7) demonstrate an interesting feature that the increase in EPSC amplitude as well as 20-80% rise-time associated with the rise in cleft heterogeneity conditions is more prominent for lesser number of glutamate released. For instance, particularly EPSC amplitude may achieve almost 50% increase across increasing *σ*_*Dglut*_ for *N*_*T*_ = 500, which is a significant amount. Also, an important feature to be noted is that the profile of variation in the rise-time across *σ*_*Dglut*_ shifts to lower values with increase in *N*_*T*_. This observation is in accordance with the earlier finding [18] which suggests that increase in the EPSC amplitude achieved through increase in the quantity of glutamate released is associated with the decrease in the 20-80% rise-time of the EPSC. At the same time, the variations in EPSC amplitude and 20-80% rise-time in response to varying *σ*_*Dglut*_ (Fig 7E) exhibit a linear relationship under all parametric conditions of *N*_*T*_. This illustrates that the strong positive correlation between these two features of EPSC is a remarkable aspect of cleft heterogeneity-mediated effects.

**Fig 8:**
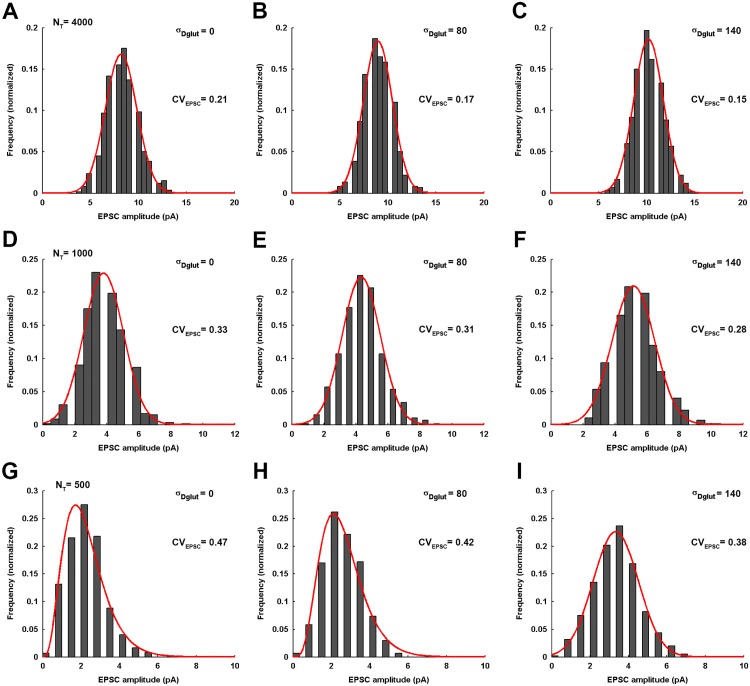
Distribution of EPSC amplitude for varying σ_Dglut_ under the different conditions of N_T_. (A-C) demonstrate the distribution of EPSC amplitudes across an ensemble of 600 independent events of the stochastic simulation of AMPARs in response to the instantaneous release of 4000 glutamate molecules at the center of the presynaptic face and for the individual values of *σ*_*Dglut*_. Their corresponding values of *CV*_*EPSC*_ are also mentioned. The EPSC amplitudes are always found to exhibit a Gaussian distribution (fitting correlations; *r*^2^ = 0.97 for *σ*_*Dglut*_ = 0, *r*^2^ = 0.98 for *σ*_*Dglut*_ = 80, and *r*^2^ = 0.98 for *σ*_*Dglut*_ = 140) across the entire range of *σ*_*Dglut*_. Similarly, (D-F) illustrate the EPSC amplitude distributions for the case of *N*_*T*_ = 1000 and for the different values of *σ*_*Dglut*_. Here also, the EPSC amplitudes are found to always exhibit a Gaussian distribution (fitting correlations; *r*^2^ = 0.97 for *σ*_*Dglut*_ = 0, *r*^2^ = 0.99 for *σ*_*Dglut*_ = 80, and *r*^2^ = 0.98 for *σ*_*Dglut*_ = 140) across *σ*_*Dglut*_. However, (G-I) demonstrate an entirely different situation for extremely low *N*_*T*_ = 500, where the EPSC amplitudes exhibit a prominent gamma-distribution for the initial lower values of *σ*_*Dglut*_ (fitting correlations; *r*^2^ = 0.97 for *σ*_*Dglut*_ = 0 and *r*^2^ = 0.98 for *σ*_*Dglut*_ = 80) but gradually acquire Gaussian-distribution at a very high *σ*_*Dglut*_ (fitting correlation; *r*^2^ = 0.99 for *σ*_*Dglut*_ = 140). Notably, the *CV*_*EPSC*_ rises significantly for any given value of *σ*_*Dglut*_ as the *N*_*T*_ is reduced. It is stated that all the histogram profiles of EPSC amplitude distributions under the different conditions discussed here have been constructed using the Freedman-Diaconis rule, which serves as a consistent basis of deciding bin-size.

**Fig 9:**
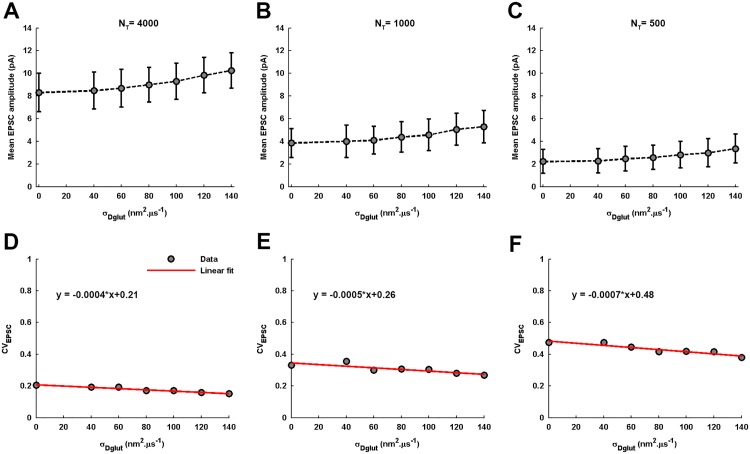
Variations in mean EPSC amplitude and CV_EPSC_ across σ_Dglut_ under the different conditions of N_T_. (A-C) demonstrate that the mean EPSC amplitude gradually increase with the rise in *σ*_*Dglut*_ under all conditions of *N*_*T*_. However, the entire profile of variation shifts to significantly lower values as the *N*_*T*_ decreases. On the contrary, (D-F) show that the *CV*_*EPSC*_ gradually decreases with increase in *σ*_*Dglut*_ under all conditions of *N*_*T*_ and the entire profile of this variation shifts to higher values as the *N*_*T*_ is reduced. Although the slopes of the linear fittings to the *CV*_*EPSC*_ variations appear extremely small owing to the large differences in the scales of the quantities associated with the x and y axes, the decrease in the noisiness of EPSC across the *σ*_*Dglut*_ range is 26.9%, 21% and 19.4% for the *N*_*T*_ = 4000, 1000 and 500, respectively, obtained from the estimates of the corresponding linear fittings.

Furthermore, the numerical treatment reveals that, for higher values of *N*_*T*_, the EPSC amplitude always exhibits a Gaussian-distribution across an ensemble of identically-parameterized stochastic activations of AMPARs (Fig 8). However, for very low *N*_*T*_, the EPSC amplitude follows a gamma-distribution for lower values of *σ*_*Dglut*_ and seems to gradually acquire more of a Gaussian-distribution profile as the *σ*_*Dglut*_ is increased. Moreover, mean EPSC amplitude gradually rises and *CV*_*EPSC*_ gradually decreases with increase in *σ*_*Dglut*_ under all circumstances of *N*_*T*_ (Fig 9). Notably, the profile of *CV*_*EPSC*_ variation shifts to higher values with decrease in *N*_*T*_, owing to the higher fluctuations in the glutamate concentration within the local volume above the PSD. Another feature of importance can be observed through the amount of decrease in *CV*_*EPSC*_ across the entire range of *σ*_*Dglut*_ as the *N*_*T*_ is reduced, given that there occurs approximately 27%, 27%, 21% and 19.4% decrease in *CV*_*EPSC*_ for *N*_*T*_ = 4000, 2000, 1000 and 500, respectively. It seems that the effectiveness of rising cleft heterogeneity for reducing the noise content of EPSC generation suffers a decline as the *N*_*T*_ is reduced. However, if we look at the slopes of the linear fittings, the rate of decrease in *CV*_*EPSC*_ across *σ*_*Dglut*_ is faster for lesser *N*_*T*_ and illustrate larger impact of cleft heterogeneity on EPSC noise. Therefore, the lesser percentage decrease for lower *N*_*T*_ is merely due to the fact that reducing *N*_*T*_ inherently brings higher fluctuations in EPSC.

**Variation in r_0_.** Here, *r*_0_ is varied while keeping rest of the parameters identical to the standard setup. Accordingly, to investigate the effect of cleft heterogeneity on the consequent EPSC generation analytically, a new set of equations are required to capture the spatially-averaged glutamate transients *C*_*glut*_(*t*) in the absence as well as presence of *σ*_*Dglut*_ in a manner identical to the previously dealt situation of *r*_0_ = 0*nm*, given as,

$$\begin{array}{ccc} C(t) & = & \frac{2N_{T}}{\pi L\rho \varepsilon }\sum_{n=1}^{+\infty }\sum_{m=-\infty .m\neq 0}^{+\infty }\frac{J_{0}\left(k_{n}r_{0}\right)J_{1}\left(k_{n}\rho \right)}{\lambda_{0n}J_{1}^{2}\left(\lambda_{0n}\right)}\frac{{\left(-1\right)}^{m}}{m\pi }\sin\left(\eta_{m}\varepsilon \right)e^{-(k_{n}^{2}+\eta_{m}^{2})Dt}\\ & & +\frac{2N_{T}}{\pi L\rho h}\sum_{n=1}^{+\infty }\frac{J_{0}\left(k_{n}r_{0}\right)J_{1}\left(k_{n}\rho \right)}{\lambda_{0n}J_{1}^{2}\left(\lambda_{0n}\right)}e^{-(k_{n}^{2}Dt)} \end{array}$$ (8)

$$\begin{array}{ccc} C^{rnd}\left(t\right) & = & \frac{2N_{T}}{\pi L\rho \varepsilon \theta^{k}}\sum_{n=1}^{+\infty }\sum_{m=-\infty ,m\neq 0}^{+\infty }\frac{J_{0}\left(k_{n}r_{0}\right)J_{1}\left(k_{n}\rho \right)}{\lambda_{0n}J_{1}^{2}\left(\lambda_{0n}\right)}\frac{{\left(-1\right)}^{m}}{m\pi }\sin\left(\eta_{m}\varepsilon \right){\left(\left(k_{n}^{2}+\eta_{m}^{2}\right)t+\frac{1}{\theta }\right)}^{-k}\\ & & +\frac{2N_{T}}{\pi L\rho h\theta^{k}}\sum_{n=1}^{+\infty }\frac{J_{0}\left(k_{n}r_{0}\right)J_{1}\left(k_{n}\rho \right)}{\lambda_{0n}J_{1}^{2}\left(\lambda_{0n}\right)}{\left(k_{n}^{2}t+\frac{1}{\theta }\right)}^{-k} \end{array}$$ (9)

**Fig 10:**
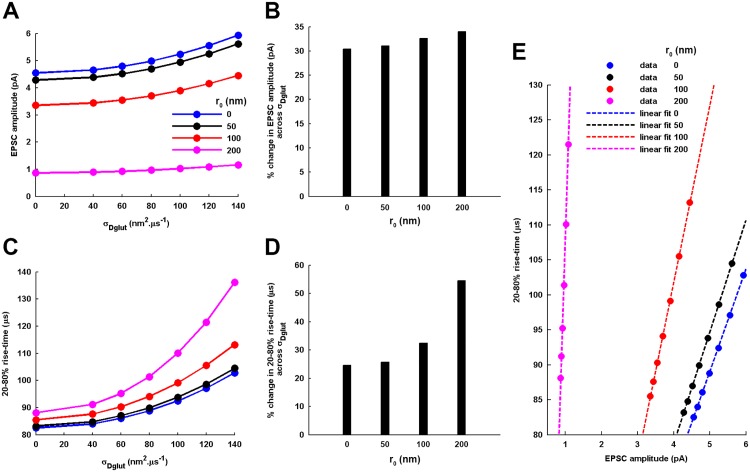
Dependency of the cleft heterogeneity-mediated tuning of EPSC amplitude and 20-80% rise-time on the presynaptic radial position, *r*_0_, of transmitter release. Accordingly, the other parameters of the synapse model are kept identical to that considered under the standard framework previously-investigated. (A) and (C) demonstrate the effect of considering different *r*_0_ on the profile of increase in EPSC amplitude and 20-80% rise-time across increasing *σ*_*Dglut*_. As evident, the amplitude and rise-time profiles shift to lower and higher values, respectively, as the *r*_0_ is increased. (B) and (D) provide the percentage change in the EPSC amplitude and 20-80% rise-time, respectively, across the entire range of increasing *σ*_*Dglut*_ for the different values of *r*_0_. (E) demonstrate that the variations in EPSC amplitude and rise-time always follow a strict linear relationship for all values of *r*_0_ (linear fittings: *y* = 14.624 ∗ *x* + 15.918 for *r*_0_ = 0, *y* = 15.982 ∗ *x* + 14.753 for *r*_0_ = 50, *y* = 25.311 ∗ *x* + 0.51854 for *r*_0_ = 100, and *y* = 161.91 ∗ *x* − 53.786 for *r*_0_ = 200 *nm*) and, thus, asserts a characteristic property of the cleft heterogeneity-mediated tuning of the two EPSC features.

As evident from the analytical results (Fig 10), the amount of increase in the amplitude and 20-80% rise-time of the EPSC profile across *σ*_*Dglut*_ is higher for more radially-dislocated sites of presynaptic vesicle-fusion events. However, the impact on 20-80% rise-time is found to be much stronger than that on the EPSC amplitude. Further, the profile of variation in the EPSC amplitude and rise-time across *σ*_*Dglut*_ shifts to lower and higher values, respectively, as the increase in *r*_0_ leads to the building of smaller local glutamate *C*_*glut*_(*t*) and extends the time of reaching peak *C*_*glut*_(*t*) since the moment of vesicle-fusion. Also, the variations in EPSC amplitude and rise-time in response to varying *σ*_*Dglut*_ exhibit a linear relationship (Fig 10E) for all conditions of *r*_0_. Therefore, like the case of variation in *N*_*T*_, this observation re-asserts it as a characteristic property of cleft heterogeneity-mediated tuning of the EPSC.

**Fig 11:**
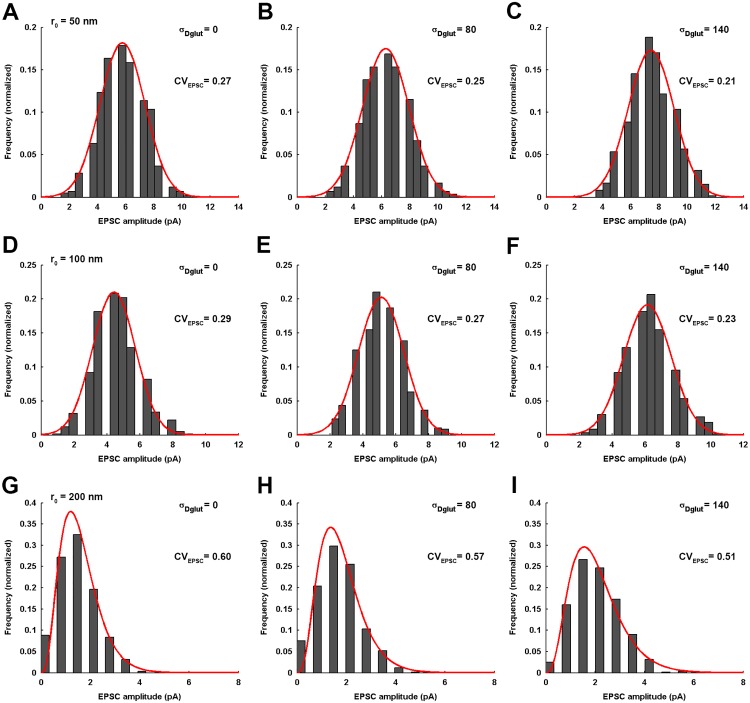
Distribution of EPSC amplitude for varying σ_Dglut_ under the different conditions of r_0_. (A-C) demonstrate, for the different values of *σ*_*Dglut*_, the EPSC amplitude distributions across an ensemble of 600 independent events of the stochastic simulation of AMPARs in response to the instantaneous release of 2000 glutamate molecules but at a radial position *r*_0_ = 50*nm* away from the center of the presynaptic face. Their corresponding values of *CV*_*EPSC*_ are also mentioned. The EPSC amplitudes consistently exhibit a Gaussian distribution (fitting correlations; *r*^2^ = 0.97 for *σ*_*Dglut*_ = 0, *r*^2^ = 0.99 for *σ*_*Dglut*_ = 80, and *r*^2^ = 0.96 for *σ*_*Dglut*_ = 140) as the *σ*_*Dglut*_ is varied. Similarly, (D-F) illustrate the EPSC amplitude distributions for the case of *r*_0_ = 100*nm* and for the different values of *σ*_*Dglut*_, where the EPSC amplitudes are again found to exhibit a Gaussian distribution (fitting correlations; *r*^2^ = 0.97 for *σ*_*Dglut*_ = 0, *r*^2^ = 0.97 for *σ*_*Dglut*_ = 80, and *r*^2^ = 0.98 for *σ*_*Dglut*_ = 140) regardless of the magnitude of *σ*_*Dglut*_. However, (G-I) demonstrate that, for extremely large *r*_0_ = 200*nm* where the glutamate release occurs significantly away from the PSD region and close to the glial absorbing boundary, the EPSC amplitudes consistently exhibit a prominent gamma-distribution for all values of *σ*_*Dglut*_ (fitting correlations; *r*^2^ = 0.97 for *σ*_*Dglut*_ = 0, *r*^2^ = 0.97 for *σ*_*Dglut*_ = 80, and *r*^2^ = 0.99 for *σ*_*Dglut*_ = 140). Notably, the *CV*_*EPSC*_ rises significantly for any given value of *σ*_*Dglut*_ as the *r*_0_ is increased. It is stated that all the histogram profiles of EPSC amplitude distributions under the different conditions discussed here have been constructed using the Freedman-Diaconis rule, which serves as a consistent basis of deciding bin-size.

**Fig 12:**
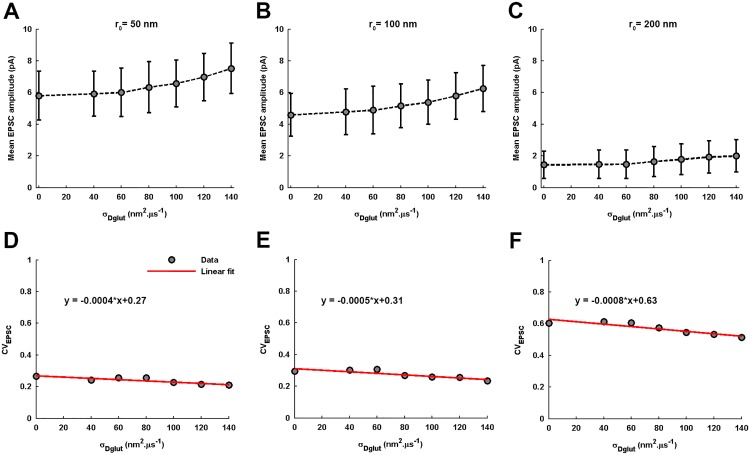
Variations in mean EPSC amplitude and CV_EPSC_ across σ_Dglut_ under the different conditions of r_0_. (A-C) demonstrate that the mean EPSC amplitude gradually increase with the rise in *σ*_*Dglut*_ under all conditions of *r*_0_. However, the entire profile of variation shifts to significantly lower values as the *r*_0_ is increased. On the other hand, (D-F) show that the *CV*_*EPSC*_ gradually decreases with increase in *σ*_*Dglut*_ under all conditions of *r*_0_ and the entire profile of this variation shifts to higher values as the *r*_0_ is increased. Although the slopes of the linear fittings to the *CV*_*EPSC*_ variations under all *r*_0_ conditions appear extremely small owing to the large differences in the scales of the quantities associated with the x and y axes, the decrease in the noisiness of EPSC across the *σ*_*Dglut*_ range is 21%, 22.1% and 17% for the *r*_0_ = 50, 100 and 200 *nm*, respectively, obtained from the estimates of the corresponding linear fittings.

Furthermore, the numerical results demonstrate that, for smaller *r*_0_ (such as *r*_0_ = 50-100*nm*), the EPSC amplitude always exhibits a Gaussian-distribution across an ensemble of identically-parameterized stochastic activations of AMPARs (Fig 11). However, for very large *r*_0_ such as *r*_0_ = 200*nm*, the EPSC amplitude follows a very prominent gamma-distribution over the entire range of *σ*_*Dglut*_. Moreover, mean EPSC amplitude gradually rises and *CV*_*EPSC*_ gradually decreases with increase in *σ*_*Dglut*_ under all circumstances of *r*_0_ (Fig 12). Notably, the profile of *CV*_*EPSC*_ variation across *σ*_*Dglut*_ shifts to higher values with increase in *r*_0_, owing to the higher fluctuations in the local glutamate concentration as the site of release is dislocated more away from the center of the PSD. Again, in this case, an approximate decrease of 27%, 21%, 22.1% and 17% in *CV*_*EPSC*_ for *r*_0_ = 0, 50, 100 and 200*nm*, respectively, across the range of increasing *σ*_*Dglut*_ suggests at first glance that the effectiveness of rising cleft heterogeneity in reducing the noise content of EPSC generation suffers a decline as the *r*_0_ is increased. However, the increasing slopes of the linear fittings demonstrate that the rate of decrease in *CV*_*EPSC*_ across *σ*_*Dglut*_ is faster for larger *r*_0_. It must be noted that larger radial deviation of release site from the center of presynaptic face inherently leads to higher fluctuations in EPSC.

The gamma-distribution of the EPSC amplitudes at a radial dislocation of 200*nm* away from the center closely resembles the related findings made in the study performed by Postlethwaite et al. [43] at the rat calyx of Held synapses. However, the quantitative differences that we observe between the findings of the two studies is due to the differences in the various important parameters of the synaptic machineries, including the amount of transmitters released per vesicle-fusion event, AMPAR density, AMPAR activation schemes and the geometrical features of the CA1 synapses and the calyx of Held synapses, as well as the approach of employing uniformly distributed release sites across an ensemble of trials in the earlier study and a fixed radial position of release site investigated at a time in the present study. We would like to state here that, for a fair analysis of the profiles of EPSC amplitude distribution, the histograms under all circumstances have been consistently constructed on the basis of Freedman-Diaconis Rule.

Eventually, in light of the above observations, it is suggested that cleft heterogeneity has selectively higher impact on the synaptic response to lesser number of transmitters released and more radially-dislocated release sites. In other words, higher cleft heterogeneity may capacitate the CA1 synapse towards such events of synaptic transmission when lesser EPSC amplitude with larger noisy fluctuations are intrinsically generated. At the same time, the standard model framework, with which the present study begins, clearly demonstrates that if the released transmitter quantity is large and released more close to the center of the presynaptic active zone above the PSD, the *CV*_*EPSC*_ will be lesser as well as the EPSC amplitudes would exhibit a Gaussian distribution. This observation has also been implied in the earlier theoretical studies performed by Freche et al. [4] and Trommershäuser et al. [16]. Therefore, the standard framework indeed points towards the amount of transmitter released by a vesicle-fusion event and the presynaptic site of transmitter release as two very important additional sources of variability, given a fixed AMPAR density for an individual CA1 synapse. These factors are typically captured under the natural conditions of the experimental investigations in a collective manner and may together lead to high EPSC variability as well as a gamma-distribution profile of EPSC amplitudes.

## Discussion

### Origin of cleft heterogeneity and the nature of retardation of glutamate diffusion

In the study involving high-pressure freezing of CA1 hippocampal slices from postnatal P21 rats [9], a highly dense material in the cleft space is observed. A similar observation is obtained in the electron microscopy with ethanol phosphotungstic acid (EPTA) staining [44] of synapses in the nucleus tractus solitari of newly born rats at different stages of development. The cryo-electron microscopy study [45] demonstrates that the density of the material in cleft region is much higher than that of the cytoplasm. It also shows the periodic organization of numerous trans-synaptic fibrils present in the cleft space. Examination of the structural composition of the synaptic cleft at cerebellar excitatory synapses using electron microscopy and freeze-substitution technique [8] reveals the presence of dense bridging fibrils, which span the cleft region providing a mechanical role in holding both the synaptic membranes together, numerous fine fibrils running parallel to the membranes, and columnar pegs protruding out of the postsynaptic membrane into the cleft space. Even the presence of extracellular matrix components abundantly contributes to the macromolecular crowding within the cleft space [10].

Moreover, the study of the three-dimensional architecture of the presynaptic cytomatrix performed in the CA1 area of rat hippocampus using electron tomography [46] provides images which clearly demonstrate irregular surface of the pre- and post-synaptic membranes confining the synaptic cleft. A more prominent evidence of irregular geometry of synaptic cleft is provided by the study involving three-dimensional reconstruction of CA1 synapses in rat hippocampal area [12]. A similar observation is provided by another study on the three dimensional reconstruction of dendritic spines and the synaptic contact made by the presynaptic terminals on the spines [11]. These and many more such evidences emphasize on the irregular cleft volume in which the glutamate diffusion takes place.

Intermittent steric and electrostatic interactions of diffusing glutamate molecules with the presynaptic as well as postsynaptic membranes [47, 48], the long-running horizontal as well as transynaptic fibrils [41, 49], and other macromolecular proteins and glycoproteins [10] in the extracellular space of cleft constantly affect the trajectory of glutamate diffusion. Except a small section of macromolecular crowd which may exhibit a regular organization [45], majority of extracellular molecules do not reside in a uniform manner in the cleft space [50]. Under such a condition, the glutamate would not be able to diffuse uniformly in the cleft space. Accordingly, the macromolecular crowding in the cleft is prominently heterogeneous and together with the irregular cleft geometry leads not only to excessive retardation of glutamate diffusion but also to significant spatial variation in the *D*_*glut*_ across the cleft space.

In fact, retarded diffusion of glutamate has already been noted well. A recent study of the spatiotemporal profile of glutamate concentration in the cleft region at calyx of Held synapse in postnatal rats [7] has shown that the effective *D*_*glut*_ in synaptic cleft is 0.3*μm*^2^.*ms*^−1^, almost two-and-a half times smaller than that in free aqueous solution, which is 0.75*μm*^2^.*ms*^−1^ [51]. Apparent *D*_*glut*_ in synaptic cleft ranging from 0.2-0.4*μm*^2^.*ms*^−1^ has been reported in the study involving patch clamp recording of excitatory current and modeling of glutamate diffusion at mossy fiber-granule cell synapses found in cerebellum [5]. Altogether, it is observed that in the synaptic cleft, there occurs almost two to four times reduction in the effective *D*_*glut*_ compared to that in free aqueous medium. An important fact to be noted here is that, although the reduced effective *D*_*glut*_ captures the overall retarded diffusion of glutamate, the role of cleft heterogeneity and, thus, the exact nature of retardation remains obscure under the simplifying situation of a homogeneous cleft medium with low glutamate diffusivity. Undoubtedly, this simplified approach has indeed served to a great extent in our understanding of synaptic transmission so far. However, it also becomes important to know how this effective *D*_*glut*_ comes forth from a more detailed nature of diffusion retardation.

The heterogeneity in cleft medium is expected to cause anomalous sub-diffusion of glutamate. In this regard, the study performed by Lacks [52] on the diffusion of acetylcholine at neuromuscular junction has highlighted the role of anomalous diffusion behind the retardation of diffusion. This study has also emphasized on the difference between the role of two prominent factors viz. tortuosity and anomalous diffusion, in the retardation of neurotransmitter diffusion. However, the major source of anomalous diffusion described in that study is the geometrical irregularity of cleft, particularly the numerous folds on the postsynaptic membrane of the neuromuscular junctions. Therefore, the present study deals with a more detailed mechanism of the retardation of glutamate diffusion which considers the spatial heterogeneity in the cleft medium owing to both the macromolecular crowding and geometrical irregularity at the cleft. Using a first-order approximation to anomalous diffusion based on the superstatistics, it shows how crucially the cleft heterogeneity shapes the glutamate diffusion profile and the resulting generation of postsynaptic EPSCs.

**Fig 13:**
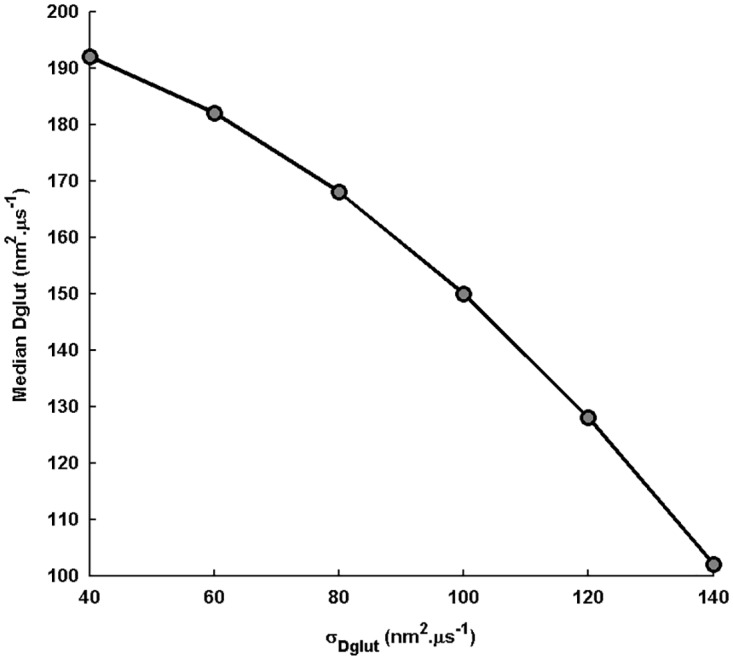
Variation in the median of the D_glut_ distribution with change in σ_Dglut_. Increase in the *σ*_*Dglut*_ of the *D*_*glut*_ distribution, while keeping the <*D*_*glut*_> fixed at 200*nm*^2^.*μs*^−1^, causes a significant decrease in the median *D*_*glut*_. This decrease has an important consequence when the median *D*_*glut*_ essentially features the effective *D*_*glut*_ used in the standard diffusion description of the glutamate transients.

Further, since spatial fluctuation in *D*_*glut*_ seems strongly possible under the experimentally-observed cleft composition, based on the present study, we suggest that glutamate diffusion always exhibits a power law behavior. At the same time, the typically-used effective *D*_*glut*_ for the standard diffusion description may correspond to the median *D*_*glut*_ in the gamma distribution profile (Fig 1C). It should be noted that, in this way, effective *D*_*glut*_ is different from and lower than the <*D*_*glut*_> of the distribution. A concomitant decrease in the magnitude of median *D*_*glut*_ with increase in the *σ*_*Dglut*_ (Fig 13) demonstrates that the slowing down of the temporal characteristics of the glutamate transient with increase in *σ*_*Dglut*_ shown here can be captured, to some extent, by the decrease in the effective *D*_*glut*_ for the standard diffusion of glutamate. However, it is straightforward that many important properties obtained from the detailed approach would indeed be missing under this approximation. Nonetheless, the effective *D*_*glut*_ may resemble the <*D*_*glut*_> only if the spatial fluctuation is sufficiently narrow. Under such conditions, the large power law exponent would also lead to *C*_*glut*_(*t*) roughly approximated by the standard diffusion profile. The study performed by Nielsen et al. (2004) involving a controlled application of dextran as a crowding agent in the cleft space at MF-GC synapses to modulate glutamate diffusion provides a strong experimental support to the effect of varying the intensity of cleft heterogeneity on EPSC amplitude and kinetics demonstrated in the present study. In fact, the results shown in that study relies on the description of effective diffusion coefficient and can, thus, be explained on the basis of the median *D*_*glut*_ mentioned here.

Accordingly, statistical properties of cleft *D*_*glut*_ distribution may, thus, also appear as an additional factor of variability across nearly identical synapses, which share almost similar necessary factors of the synaptic response except the intensity of cleft heterogeneity among them. In this regard, the precise dendritic recordings of EPSC generation at the CA1 synapses performed by Magee and Cook [42] provide an important experimental observation which may be adequately explained on the basis of the findings of the present study. In fact, the present study involves a formulation of the synaptic transmission using the appropriate experimental estimates of the various parameters of the CA1 synapses only. In their study, it has been observed that the mean EPSC amplitude as well as the rise-time kinetics of the smallest dendritic events gradually increases while going from proximal to distal synapses on the dendrites of CA1 neurons. Accordingly, it has been emphasized that unitary events with larger amplitude and slow rise-time kinetics are prevalent at distal synapses. Although this increase follows a statistically consistent profile, it is considerably small so that the known prominent processes in the synaptic adaptation cannot be accounted for this observation and, hence, has remained unanswered clearly. However, the profiles of variation in the mean EPSC amplitude and 20-80% rise-time kinetics obtained here across the increasing cleft heterogeneity under the standard setup of investigation not only reflect a very similar small resultant increase but also the profile of mean EPSC amplitude closely resembles the earlier observed profile in a quantitative manner. Therefore, variation in the cleft crowding conditions from proximal to distal synapses is suggested to be a possible factor behind the experimentally-observed variations in EPSC generated under smallest dendritic events across the synapses.

### A possible secondary role of cleft heterogeneity in tuning EPSC during synaptic plasticity

Certainly, if the cleft heterogeneity has a potential in modulating the postsynaptic EPSCs, one may always expect its possible involvement in the temporal modifications at a single individual synapse under the phenomenon of plasticity. In this regard, the present observations clearly suggest that, under all circumstances, the quantitative impact of variation in cleft heterogeneity on the various features of the EPSC remains significantly smaller than that exerted by changes in the well-know prominent regulating factors involved in the synaptic plasticity [4, 18]. However, there are certain important aspects of the effect of cleft heterogeneity which cannot be overlooked. Recognizing the fact that there exists a tight constraint on the metabolic cost of maintaining the functioning of neural circuitry under the real physiological scenario [53–55], the magnitudes of increase in the EPSC amplitude and decrease in its noise due to the increase in cleft heterogeneity cannot be underestimated as it is cost-effective and occurs merely through a physical process of obstructing glutamate diffusion. The other very important feature is that cleft heterogeneity has a selectively higher impact on the events of less transmitter release and more radially-dislocated sites of release with respect to the PSD center. Therefore, higher cleft heterogeneity can be an effective secondary means of enhancing the synaptic response even towards such events of presynaptic transmitter release which otherwise intrinsically lead to weaker and more noisy EPSCs.

Nonetheless, an important question that lies in the way is how cleft heterogeneity could be regulated during plasticity. At this point, it must be recognized that the proposed simplified mathematical model in the present study does not emphasize on the exact spatial variation in the diffusivity of glutamate lying across a synaptic cleft space. For obvious reasons, this is seemingly irreproducible and beyond any directed regulation achievable along the route of gaining synaptic plasticity. Rather, what we understand as the strong point of the model is that it insists on the statistical aspect of diffusivity prevailing across cleft space which may involve a regulatory hand and may bear a consistency across synapses with different levels of plasticity. Therefore, the emerging question is how the statistical properties of glutamate diffusivity across the cleft space may be regulated.

For the present context, the various components of the macromolecular crowding in the cleft space may be sorted into two groups: One which is comprised of the typical prominent architecture of the excitatory synapses such as cell adhesion proteins (N-Cadherins, Catenins and Neuroligins-Neurexins), glutamate-binding AMPA and NMDA receptors and other essential transmembrane proteins including RTKs, which protrudes out into the cleft space and engenders a significant fraction of crowding in the cleft space [56] whereas the other group involves the components of the extracellular matrix (ECM) [10]. The density and spatial distribution of the architectural components are very tightly regulated under the process of synaptic plasticity. On the other hand, the composition of ECM is itself dynamic in nature [10]. Also, a major fraction of the recognized repertoire of ECM molecules (reelin, integrin and laminin) shows strong specific interactions with the various architectural components and exhibit clustering of ECMs in the cleft space around their interacting transmembrane partners [10, 50]. Therefore, it is strongly possible that the specific and preferred spatial accumulation of the different ECMs around the tightly regulated architectural components of a synapse may together give rise to statistically consistent properties of the resultant crowding intensity and heterogeneity. Even if geometrical irregularity is neglected to have any directed regulation, the macromolecular crowding alone is sufficient to cause the major amount of spatial heterogeneity in the glutamate diffusivity.

Interestingly, the suggested mechanism of regulation of cleft crowding properties itself comes forth as an interesting aspect of investigation. At least, a pertinent computational experiment that we expect to alleviate this dilemma to some extent in future is to scale the distribution of different architectural components and the pattern of crowding of individual ECM, as far as possible, across the cleft space through their known interactions with the transmembrane elements of the synapse protruding out in the cleft. Subsequently, using biophysical simulation of glutamate diffusion considering in detail the various steric and electrostatic interactions of the diffusing glutamate molecules with these crowding elements may provide an insight into how the statistics of diffusivity across the cleft space may emerge.

These facts together indicate that cleft heterogeneity may serve as a secondary role in plasticity. Accordingly, the present study sheds light on an additional way through which dynamic composition of the architectural elements of a CA1 synapse and the associated ECM during plasticity may shape the features of quantal response as a sideways mechanism by eventually altering not only the mean *D*_*glut*_ but also the other distribution properties of *D*_*glut*_ including *σ*_*Dglut*_ and median *D*_*glut*_. An important point to note here is that, since the plasticity-associated modifications of synapse occur at a timescale from minutes to hours and glutamate diffusion occurs at submillisecond timescale, a single event of presynaptically-released glutamate diffusion always experiences a static statistical distribution of *D*_*glut*_ prevailing across the cleft at the time of release event.

## Conclusion

In the present study, we show how the spatial heterogeneity in the macromolecular crowding and sudden geometrical irregularities in the cleft space may affect the glutamate transients in the cleft and the generation of EPSCs. The new framework of glutamate diffusion is, in fact, a one-step improvement over the conventionally-used standard diffusion framework which facilitates the realization of the prime objectives of the present study. Cleft heterogeneity is seen to cause a power law behavior in the glutamate transients, which suggests long-range anomalous subdiffusion of glutamate in the cleft. Moreover, the change in amplitude and temporal characteristics of the EPSC with variation in the intensity of cleft heterogeneity suggests a possible secondary role of cleft heterogeneity in tuning the synaptic transmission during homeostasis as well as synaptic plasticity.

Nonetheless, the peak glutamate concentration in the entire cleft space obtained in the present theoretical study closely resembles the earlier recorded peak glutamate concentration in the CA1 synaptic clefts under single vesicle release [2, 5, 34]. Moreover, the range of mean EPSC amplitudes recorded here under different conditions of *D*_*glut*_ distribution is fairly close to the range of mean EPSC amplitudes of smallest dendritic events experimentally recorded at the CA1 dendrites by Magee and Cook [42]. The quantitative resemblance assures the close enough calibration of the various parameters associated with the glutamate diffusion, synapse geometry, and AMPAR activation performed in the present study.

Although other potential factors such as the simultaneous fluctuations in transmitter content released and transmitter release position across a train of presynaptic vesicle-fusion events, geometry of the cleft, density of AMPARs etc. have not been altered here to satisfy the main objective of the present work, their variation along with the factor of cleft heterogeneity may exhibit further significant features of synaptic transmission. Nonetheless, in light of the above observations, it remains important to experimentally enquire about the cleft heterogeneity pertinent to glutamate diffusion at excitatory synapses and its effects on the synaptic response. Notably, the detailed measurement of the physical quantities of interest of the glutamate diffusion within the synaptic cleft space has still remained a daunting challenge. Although many advancements in the knowledge of chemical (drugs and neurotransmitters) diffusion at the brain tissue level has been achieved by now, the diffusion of glutamate at the highly constricted cleft dimension of nanometer scale remains an exception [14, 38]. Although many ingenious technical designs such as high-affinity receptors, glutamate EOS [57, 58], GluSnFR etc. have been realized so far, there are practical limitations to visualize/measure glutamate diffusion within cleft space to great details [14]. Nonetheless, many more advanced techniques such as FLIM, FCS, sptPALM, TR-FAIM etc., together with the earlier designs, are under improvisations to gain an access to the physically-constrained space of synaptic cleft and to provide information regarding glutamate diffusion at the desired spatiotemporal resolutions. Therefore, it is realized that the primary subject of the present research work is still in its extreme infancy. However, the proposed formulation serves as a simplified but effective theoretical means to capture the effect of cleft heterogeneity and stands as a rational basis to demand for a future experimental enquiry.
